# Clinical Significance of Hepatobiliary Localization of Tc-99m EC in Diuretic Renography

**DOI:** 10.1055/s-0044-1779748

**Published:** 2024-04-22

**Authors:** Deepa Singh, Sanchay Jain, Anuj Jain, Suruchi Jain

**Affiliations:** 1 Department of Nuclear Medicine, All India Institute of Medical Sciences (AIIMS), Bhopal, Madhya Pradesh, India; 2 Department of Anaesthesiology, All India Institute of Medical Sciences (AIIMS), Bhopal, Madhya Pradesh, India

**Keywords:** renal dynamic scintigraphy, hepatobiliary excretion of Tc-99m EC, bowel, gallbladder and/or liver visualization on Tc-99m EC scan, SPECT/CT

## Abstract

**Objective** Technetium-99m ethylene dicysteine (Tc-99m EC) is a well-established, tubular tracer for diuretic renography. Few occasional cases have been reported in literature regarding visualization of liver, gallbladder (GB), or bowel due to increased hepatobiliary route of excretion of Tc-99m EC on diuretic renography. This study aimed to retrospectively review the incidence of visualization of liver, GB, or bowel and its clinical significance in Tc-99m EC diuretic renography.

**Materials and Methods** Data of all patients who underwent diuretic renography in the department from January 24, 2022 to March 31, 2023 was included in the study. The data was analyzed to assess the incidence of visualization of GB or bowel loops, correlation of the hepatobiliary localization with factors like age of the patient, concentration of 99m TcO4 solution, quality control parameters, presence of renal stone disease, serum creatinine, relative renal function, and effective renal plasma flow. Effect of hepatobiliary localization on scan interpretation and reporting was assessed.

**Results** The retrospective analysis of 437 diuretic renograms revealed the hepatobiliary localization of tracer in 34 patients. Out of these 34 patients, 14 patients had only faint visualization of tracer at 4 hours delayed image. Twenty scans had visualization of both GB and bowel. Out of these 20 scans, GB and bowel were visualized during dynamic imaging in one scan, after initial 20 minutes in two scans and in 2 to 4 hours delayed images in rest of the 17 scans. Two out of 20 patients had increased serum creatinine, 16 patients had either single kidney or relative renal function less than 26%, and 12 patients had renal stone disease. Out of the four patients in whom relative renal function was more than 25%, one patient had raised serum creatinine and three patients had renal stone disease. Interpretation of images was affected only in three patients, in which reporting of the scans required single-photon emission computed tomography imaging and correlation with other imaging modalities.

**Conclusion** Hepatobiliary excretion of Tc-99m EC usually does not usually affect the scan interpretation and quantitative renogram analysis, but reader should be cognizant of the potential pitfalls during scan interpretation. In this study, we reviewed the possible causes of this hepatobiliary clearance and importance of additional views and correlation with other imaging modalities to clarify the suspicion arises for accurate reporting.

## Introduction

Renal dynamic scintigraphy is a commonly used investigation to evaluate function and drainage of kidneys. The utilization of technetium-99m ethylene dicysteine (Tc-99m EC) as a tracer for renal purposes stemmed from the observation that the brain perfusion agent ethylene dicysteine dimer is rapidly eliminated through the kidneys after being metabolized into ethylenedicysteine. 1 2 Following this discovery, numerous studies have demonstrated its superiority over already existing renal agents like Tc-99m-mercaptoacetyltriglycine (Tc-99m-MAG3) and I-131 orthoiodohippurate (OIH), attributed to its enhanced excretion characteristics and image quality. Tc-99m EC boasts a robust initial extraction rate of around 70%, limited plasma protein binding (∼ 30%), and a binding to red blood cells of approximately 5 to 6%. The primary route of excretion for EC is through the kidneys, with only minor or insignificant hepatobiliary elimination. Consequently, Tc-99m EC has now emerged as the preferred radiopharmaceutical agent for evaluating renal function. 1 2 3 Tc-99m EC was initially introduced as a potential substitute for technetium-99m mercaptoacetyl-triglycine (Tc-99m MAG3) for imaging renal tubules. The excretion of Tc-99m EC through the liver and bile ducts is minimal and typically does not interfere with regular imaging interpretation. Nevertheless, it is important to consider the chance of observing the gallbladder (GB) during Tc-99m EC renogram studies, as this could resemble an ectopic kidney. 1 2 3 4

This study aimed to retrospectively review the incidence of visualization of liver, GB or bowel, extent of interference with scan findings, need of additional views, and its clinical significance in Tc-99m EC diuretic renography.

## Materials and Methods

We retrospectively evaluated 437 Tc-99m EC diuretic renograms done at our center between January 24, 2022 to March 31, 2023 for evaluation renal function and drainage.

### Preparation of Tc-99m EC

For Tc-99m EC preparation, we used predispensed sterile formulation kit (TCK-43) for reconstitution with sterile Tc-99m sodium pertechnetate eluted from molybdenum-99/Tc-99m COLTECH generator procured from Board of Radiation and Isotope Technology (BRIT). The kit consists of 1 vial each of component A (containing 40 mg of calcium/sodium glucoheptonate and 0.1 mg of stannous chloride dehydrate in freeze dried form), component B (1 mg of ethylene dicysteine in freeze dried form) and component C (1 mL of 0.5 M sodium dihydrogen phosphate solution with pH 4–5). We generated the Tc-99m EC through reconstitution of this commercially available in vitro kit as per the guidelines provided by the manufacturer. We used this labeled Tc-99m EC within 4 hours of preparation.

### Quality Control of Tc-99m EC

As part of our department's standard procedure, we assess the effectiveness of labeling for the initial vial in each fresh batch of reagents with the Quality Control Test Kit (TCA-6) procured from BRIT. We examined the purity of Tc-99m EC through thin-layer chromatography, utilizing instant thin-layer chromatography silica gel and Whatman no. 1 supports. Ethanol at 90% and saline solvents were employed for this purpose. The efficiency of labeling was determined by measurements conducted using NaI (Tl) well counter and a gamma camera detector. Quality control assessments for labeling were performed within the range of 1 to 5 hours following the labeling process.

### Procedure of Tc-99m EC Renal Dynamic Scintigraphy

For adults, a dosage of 111 to 185 MBq (3–5 mCi) of Tc-99m EC was administered, while in the case of children, the dose was adjusted in proportion to their age and weight (according to Webster's formula).

Imaging was conducted with the patient lying supine, and the gamma camera (GE Discovery 670 DR dual head single-photon emission computed tomography/computed tomography [SPECT/CT] system) was positioned posterior to the region of the patient's kidneys. Following the intravenous bolus injection of the radiopharmaceutical, a series of dynamic images were captured in 64 × 64 matrix. Initially, the frame rate was set at 2 seconds per frame for a duration of 60 seconds (total 30 frames) for flow/ perfusion, followed by 15 seconds per frame for up to 22 minutes (total 88 frames) for cortical uptake phase. Subsequently, a pixel matrix of 128 × 128 was employed for static images. The static prevoid, postvoid, the 2-hour, and 4-hour delayed images were acquired. Full syringe and empty syringe counts were taken for 10 seconds each. In some cases, lateral view, oblique view SPECT or SPECT/CT was also obtained for precise localization of tracer distribution. SPECT was acquired in 128 × 128 matrix at 15 seconds/frame in step and shoot mode, total angular range of 360 degrees, arc per detector of 180 degrees with 5-view angles (total no of views 72). CT parameters were 120 KeV tube voltage, 250 mA tube current, slice thickness of 1.25 mm, pitch 1.375:1, and speed 13.75 mm/rotation.

### Scan Interpretation

Processed and nonprocessed renal scintigraphy images were interpreted by two nuclear medicine physician independently. Differential function as percentage radiotracer uptake, effective renal plasma flow (ERPF) was assessed by drawing region of interest between 1 and 2 minutes after radiotracer administration on the dynamic images. Transit of radiotracer through the kidneys was assessed with Tmax, peak to 1/2 peak values, and evaluation of renogram curve.

### Correlation with Other Imaging

Tc-99m EC scans were correlated with available Tc-99m labeled dimercaptosuccinic acid, ultrasonography, intravenous pyelography, micturating cysto-urethrography, and CT urography reports and images. We utilize the advantage of these imaging modalities in clarification of doubt arises from visualization of extra-renal Tc-99m EC uptake.

## Results

The analysis of the 437 dynamic scintigraphy studies revealed the hepatobiliary localization of the tracer in 34 patients (18 males and 13 females, mean age: 26.0 ± 19.1 years, age range: 3–66 years). The mean value of intravenously administrated dose of Tc-99m EC was 3.9 ± 1.2 mCi (range: 1.8–5 mCi). The mean value of serum creatinine in the study population was 1.0 ± 0.8 mg/dL (range: 0.46–3.7 mg/dL).

The intensity of abnormal site of tracer uptake was classified as faint, mild (intensity less than renal radiotracer uptake), moderate if it was equal or more than renal radiotracer uptake. Out of the 34 patients, 14 patients had only faint visualization of the tracer at 4 hour delayed imaging. Twenty patients had mild-to-moderate abnormal tracer activity with visualization of both GB and bowel. Out of these 20 patients, GB and bowel were visualized during dynamic imaging in one patient, after initial 20 minutes imaging in two patients and in 2 to 4 hours delayed images in rest of the 17 patients.

Review of the patient data of these 20 patients having visualization of both GB and bowel revealed that 2 patients had raised serum creatinine, 16 patients had either one functioning kidney or relative function of less than 26%, and 12 patients had renal stone disease. Out of the four patients in whom relative renal function was more than 25%, one patient had raised serum creatinine, and three patients had renal stone disease.

**Fig. 1:**
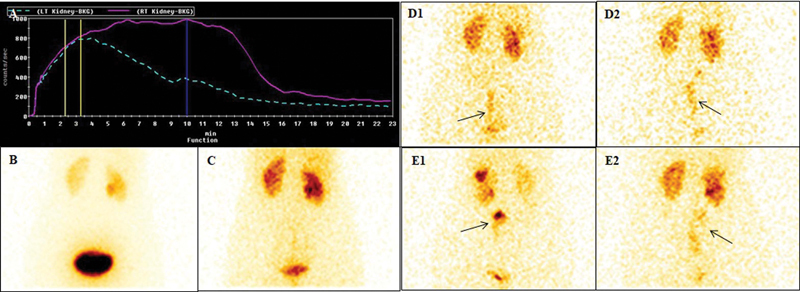
Renogram curve and static images (prevoid, postvoid, and delayed images at 2 and 4 hours) of a 34-year-old woman with a history of right pyeloplasty. Dynamic images did not reveal dilated ureter. The renogram curve was descending in both the kidneys ( **A** ). Posterior prevoid ( **B** ) and postvoid ( **C** ) static images minimal tracer in pelvicalyceal system (PCS) in left kidney and mild in right kidney. Two ( **D** ) and 4 hours ( **E** ) anterior (1) and posterior (2) static images also showed an additional abnormal site of trace uptake in the region of right ureter that is more prominent in anterior image ( *arrows* ). This was due to tracer activity in the bowel (more prominent in anterior views). Computed tomography urography also did not reveal dilated ureter. Potential pitfall here is possible perception of bowel activity as ureteric activity.

There was no correlation of hepatobiliary localization of tracer with patient's age, disease laterality, and concentration of Tc-99m solution. Interpretation of images was affected only in three patients, in which reporting of the scans required SPECT imaging and correlation with other imaging modalities ( Figs. 1 2 3 4 ).

**Fig. 2:**
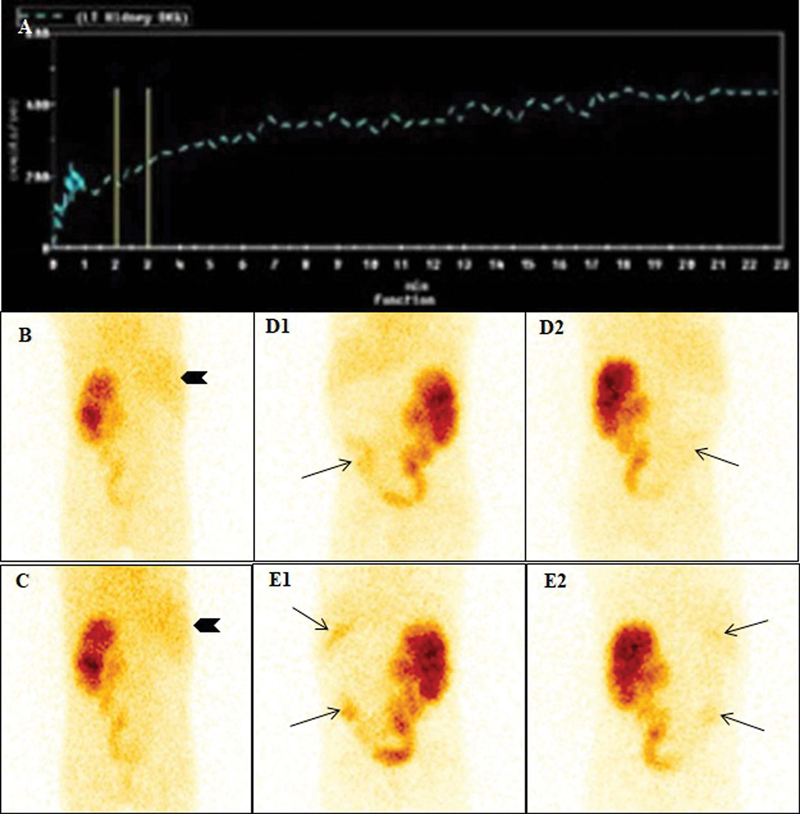
Renogram curve and static images (prevoid, postvoid, and delayed images at 2 and 4 hours) of a 5-year-old girl with enlarged and hydroureteronephrotic left kidney. Right kidney was not visualized in dynamic images. Tracer activity is noted in the right upper quadrant region and lower right renal fossa, corresponding to liver, gallbladder, and bowel activity. The renogram curve of left kidney was rising ( **A** ). The Prevoid ( **B** ) and postvoid ( **C** ) static images showed tracer activity in the PCS in the left kidney and within liver parenchyma (arrowhead). Two hours ( **D** ) anterior (1) and posterior (2) static images also showed an additional abnormal site of tracer uptake at the level of lower part of right renal fossa (arrows). Such pattern may falsely appear as faintly visualized right kidney and dilated ureter. Four hours ( **E** ) anterior (1) and posterior (2) static images showed additional abnormal sites of trace uptake at the level of lower part of right renal fossa and in the gallbladder (arrows).

**Fig. 3:**
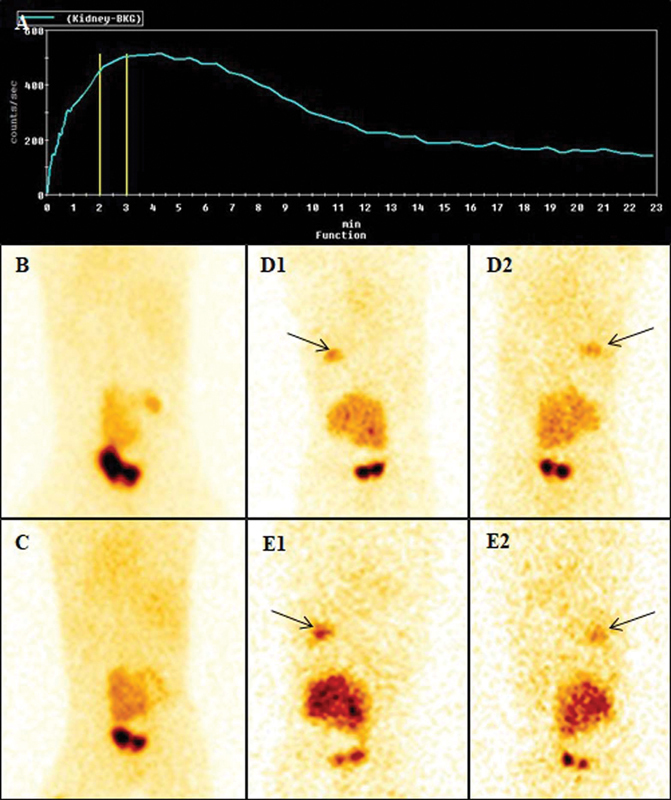
Renogram curve and static images (prevoid, postvoid, and delayed images at 2 and 4 hours) of a 3-year-old girl with pelvic pancake kidney and right pelviureteric–junction obstruction showed severely impaired perfusion and cortical tracer uptake in the right moiety of kidney. The renogram curve was descending in left moiety ( **A** ). Prevoid ( **B** ) and postvoid ( **C** ) static images showed tracer activity in the PCS in the kidney. Two hours ( **D** ) and 4 hours ( **E** ) anterior (1) and posterior (2) static images also showed an additional abnormal site of trace uptake at the level of right renal fossa ( *arrows* ). This additional site of tracer uptake in the region of right renal fossa was actually tracer uptake in the gallbladder. Such distribution in a patient with single kidney may create illusion of poorly functioning second kidney.

**Fig. 4:**
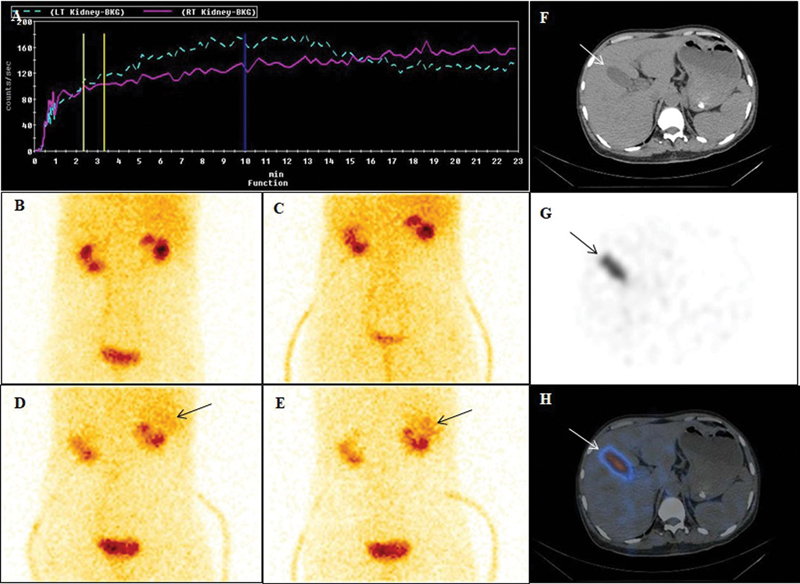
Renogram curve and static images (prevoid, postvoid, and delayed images at 2 and 4 hours) of a 45-year-old-man with bilateral nephrolithiasis. Renogram curves ( **A** ) showed imapired perfusion and cortical tracer uptake in both the kidneys. Prevoid ( **B** ) and postvoid ( **C** ) images showed small sized kidneys with impaired cortical tracer uptake. Delayed images at 2 and 4 hours ( **D** , **E** ) also showed tracer uptake along the superolateral aspect of right kidney which on single-photon emission computed tomography/computed tomography images localized to abnormal tracer uptake within the gallbladder ( **F** – **H** ) ( *arrows* ). Reporting errors may arise due to extra-renal activity in close proximity to the kidney.

Calculation of ERPF and renogram analysis was difficult in only one patient where increased background activity due to severely impaired renal function and raised serum creatinine, the delineation of kidney outline could not be done properly.

## Discussion

In this study, it is observed that there was no correlation of hepatobiliary localization of tracer with patient's age, disease laterality, and concentration of Tc-99m solution. It is usually visualized in the delayed images and usually does not affect the scan interpretation and quantitative renogram analysis. Severely impaired renal function, renal stone disease, and severely reduced relative renal function may contribute to hepatobiliary localization of the tracer.

The plasma clearance of any radiopharmaceutical occurs through diverse excretion routes. However, minimal hepatobiliary clearance of the renal agent is essential to accurately gauge the ERPF, as substantial hepatobiliary excretion could result in underestimated renal clearance values. 5 6 While I-131 orthoiodohippurate (OIH) stands as the preferred reference agent for renal clearance, technetium-based agents like MAG3 are not an optimal replacement due to their high protein binding and limited plasma clearance in humans. In contrast, Tc-99m EC demonstrates a commendable extraction fraction and renal excretion, resembling OIH in functionality. It delivers high-quality images owing to its negligible liver accumulation and a strong kidney-to-background ratio. Various authors have documented significantly reduced accumulation of EC in the liver and intestines compared with MAG3 and OIH in both animal and human studies. This diminished liver activity makes EC particularly appealing for patients with renal failure. 3 7 Prvulovich et al noted improved kidney visualization and only faint liver activity with EC in comparison to MAG3 among individuals with severe renal failure. 8 Similarly, Kibar et al found that EC images in pediatric patients exhibited superior quality when compared with MAG3 images. 9 Several factors have been identified that influence the excretion characteristics of MAG3. When patients undergoing MAG3 renal scintigraphy are not in a fasting state, there is minimal or diminished uptake in the GB. Additionally, it has been demonstrated that the presence of radiolabeled impurities is linked to the hepatobiliary excretion of MAG3. The inclusion of oxidizing agents (such as sodium hypochlorite or hydrogen peroxide) in the sodium pertechnetate solution leads to a decrease in labeling efficiency. Photolytic degradation and prolonged preparation steps for MAG3 can result in the formation of impurities. Similarly, research has indicated that reconstituting with 10 mL of saline solution yields optimal labeling efficiency, whereas using a lesser amount of saline for reconstitution leads to reduced labeling efficiency for Tc-99m MAG3. 6

Tc-99m LLEC, the end-product of the metabolic breakdown of Tc-99m LLEC-diethyl-ester (known as Tc-99m LL-ECD) employed in brain scans, is swiftly and effectively excreted through urine. Its attributes include a substantial initial extraction rate (around 70%, as opposed to 20% for Tc-99m diethylenetriaminepentaacetic acid), minimal binding to plasma proteins (roughly 30%), limited binding to red blood cells (∼ 5.7%), along with almost no clearance from areas other than the kidneys. Moreover, it exhibits a high kidney-to-background ratio. Numerous investigations in both animal models and human subjects have highlighted the markedly lower accumulation of LLEC in the liver and intestines compared with its contemporary counterparts such as MAG3/OIH. 4 10 11 12 13 14

The presence of the tracer in the cecum and ascending colon can only be accounted for by the elimination of LLEC from areas other than the kidneys. A search on PubMed yielded in a few case reports that discuss the elimination of LLEC from extrarenal sites. Arora et al detailed a case where the GB was unexpectedly visible in a 10-year-old renal transplant patient undergoing a Tc-99m EC scan. 15 Jain et al documented an occurrence where liver activity was observed in LLEC renal scintigraphy, creating a resemblance to a poorly functioning kidney within an empty right renal fossa. 16 We have conducted an analysis of the existing literature concerning LLEC and the excretion patterns of different radiopharmaceuticals, leading us to make several observations.

First, the radiochemical impurities have the potential to influence the distribution of any radiopharmaceutical, particularly when the labeling process occurs at a pH level below 12. However, in such instances, it is crucial to ensure that other concurrent cases display similar activity. Next, approximately 10% of hepatobiliary elimination has been documented for Tc-99m MAG3. Interestingly, the absence of hepatobiliary excretion presents a benefit of LLEC when compared with MAG3. Furthermore, the different stereoisomers of EC could potentially exhibit varied patterns of excretion. Also, in individuals with anuria, there have been reports of minimal hepatic uptake of LLEC (less than 6%). When kidneys are functioning normally, the hepatic uptake might not be readily noticeable due to the strong emission originating from the kidneys. 6 17 18 19 20

Administering probenecid before LLEC administration hampers the excretion of LLEC through the kidneys, leading to a decrease in the excretion of the tracer through urine. This alteration is accompanied by a more noticeable visualization of EC within the liver and a slower clearance from the bloodstream. 21 The occurrence of tracer activity within the GB has been reported in renal transplant recipient undergoing an EC renal scan. Careful assessment of the planar images is essential to avoid misinterpretation. Obtaining different planar views and SPECT ± CT may be warranted whenever necessary. 17 18 19

## Conclusion

The hepatobiliary excretion of Tc-99m EC, when present, is usually visualized in the delayed images. Although it does not usually affect the scan interpretation and quantitative renogram analysis, the reader should be cognizant of the potential pitfalls of hepatobiliary excretion of this tracer. Severely impaired renal function, renal stone disease, and severely reduced relative renal function of one of the kidneys may contribute to hepatobiliary localization of the tracer. In this study, we reviewed the possible causes of this hepatobiliary clearance and importance of additional views and correlation with other imaging modalities to clarify the suspicion arises with ultimate aim of accurate reporting that help in management of patients.
